# Emerging Role of Circular RNA–Protein Interactions

**DOI:** 10.3390/ncrna7030048

**Published:** 2021-08-04

**Authors:** Arundhati Das, Tanvi Sinha, Sharmishtha Shyamal, Amaresh Chandra Panda

**Affiliations:** 1 Institute of Life Sciences, Nalco Square, Bhubaneswar 751023, India; arundhati.s@ils.res.in (A.D.); tanvi@ils.res.in (T.S.); sharmishtha@ils.res.in (S.S.); 2 School of Biotechnology, KIIT University, Bhubaneswar 751024, India

**Keywords:** circRNA, RNA-binding protein, decoy, splicing, translation, mRNA stability

## Abstract

Circular RNAs (circRNAs) are emerging as novel regulators of gene expression in various biological processes. CircRNAs regulate gene expression by interacting with cellular regulators such as microRNAs and RNA binding proteins (RBPs) to regulate downstream gene expression. The accumulation of high-throughput RNA–protein interaction data revealed the interaction of RBPs with the coding and noncoding RNAs, including recently discovered circRNAs. RBPs are a large family of proteins known to play a critical role in gene expression by modulating RNA splicing, nuclear export, mRNA stability, localization, and translation. However, the interaction of RBPs with circRNAs and their implications on circRNA biogenesis and function has been emerging in the last few years. Recent studies suggest that circRNA interaction with target proteins modulates the interaction of the protein with downstream target mRNAs or proteins. This review outlines the emerging mechanisms of circRNA–protein interactions and their functional role in cell physiology.

## 1. Introduction

Circular RNA (circRNA) was initially discovered in viroids, followed by their discovery in eukaryotes [1,2]. For three decades, circRNAs were primarily believed to be splicing errors without much physiological relevance [3]. Recent advancements in next-generation RNA sequencing (RNA-seq) revealed that circRNAs are a large class of ubiquitously expressed covalently closed RNA molecules [4,5]. Due to their lack of poly-A tails and circular nature without free ends, circRNAs are rarely detected in the mRNA sequencing data. However, recent whole transcriptome sequencing followed by the analysis of transcriptome-wide circRNA expression using novel bioinformatics tools suggested that circRNAs are a large class of transcripts generated by backsplicing mechanisms from the pre-mRNA [4,5,6]. The covalently closed circRNAs are produced by backsplicing that ligates a downstream donor splice site with an upstream acceptor splice site [7]. CircRNAs usually range from less than a hundred to thousands of nucleotides in length [8]. CircRNAs show evolutionary conservation across the eukaryotes and show tissue- and developmental stage-specific expression patterns [5,9]. Their covalently closed structure without the free ends makes them resistant to exonucleases, making them more stable than linear mRNAs [5]. In addition, the altered expression of circRNAs during development and disease underscores their involvement in human physiology and pathology.

To date, numerous studies have established that circRNAs are abundant RNA molecules with the potential to regulate gene expression [10]. The most established mechanism of circRNA action is through acting as a sponge for microRNAs [11]. For example, *CDR1as* is the first and most well-characterized circRNA that acts as a sponge for miR-7 and regulates various cellular processes, including cancer progression, myocardial infarction, and insulin secretion [11,12,13,14]. Hundreds of reports in the last couple of years highlighted the physiological importance of circRNA by acting as a miRNA sponge [15]. Furthermore, circRNAs may interact with RBPs, which may have bidirectional effects [16]. The circRNA–RBP interaction may facilitate the biogenesis of circRNA by backsplicing that depends on the inverted repeat sequences in the flanking introns of pre-mRNA [7,16]. In addition, circRNA can regulate the target gene expression by acting as a decoy, protein sponge, and scaffold for the interacting proteins [16]. Although circRNA–protein interactions remain obscure and need further exploration, several studies have underscored their importance in cellular physiology in the last couple of years. This review aimed at providing an overview of the current knowledge of circRNA–RBP interactions and their physiological significance.

## 2. CircRNA Biogenesis and Regulatory RBPs

CircRNAs are generated by the head-to-tail joining of the exonic sequence by backsplicing [3]. Recent research into circRNA biogenesis has shown that the canonical spliceosomal machinery catalyzes backsplicing [17]. The backspliced junction is a unique characteristic of circRNAs, which plays a significant role in identifying and characterizing circRNAs. Mutated RNAP II with slower transcription speed was found to inhibit circRNA biogenesis. Another study reported increased transcription elongation rates on circRNA-producing genes compared to genes lacking circRNAs [18]. There are three main subtypes of circRNAs based on their sequence of origin. The majority of the circRNAs identified to date are exonic circRNAs (ecircRNAs) derived from one or multiple exons. At the same time, circular intronic RNAs (ciRNAs) and stable intronic sequence RNAs (sisRNAs) are derived from the introns, and exonic–intronic circRNAs (EIciRNAs) contain both exonic and intronic sequences [19].

**Figure 1:**
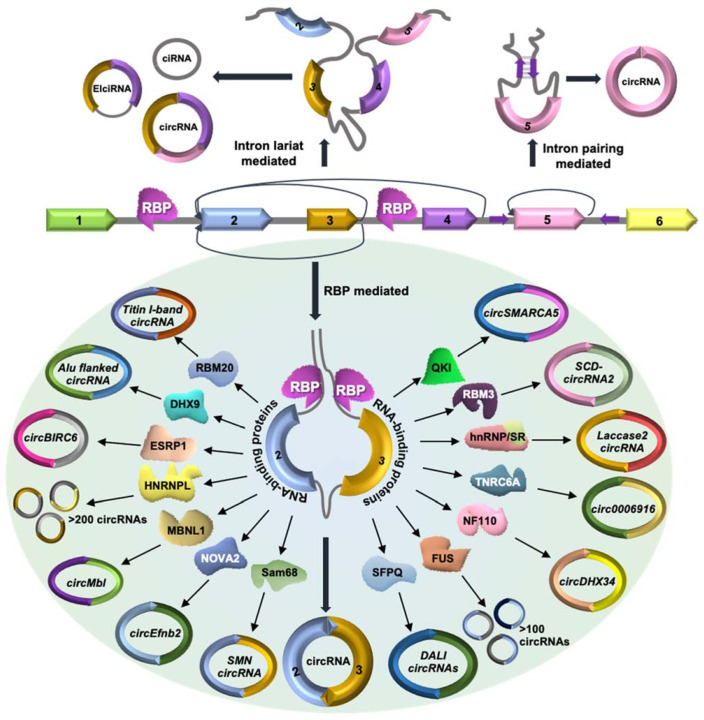
Schematic representation of circRNA biogenesis. Exonic circRNA is formed through a lariat-mediated or intron pairing-mediated backsplicing mechanism, where the downstream 5′ splice donor site of one exon covalently joins the upstream 3′ splice acceptor site. Backsplicing of circRNAs without splicing generates EIcircRNAs with a retained intron. The intronic lariats resistant to debranching generate stable ciRNAs. In addition, RBPs interacting with pre-mRNA can modulate the backsplicing of circRNAs.

The biogenesis of circRNAs has been well documented and reviewed in previous articles (Figure 1) [7]. Briefly, the formation of circRNAs can be classified into three categories. (1) Lariat-circularization or exon-skipping model: A huge lariat containing the circular RNA exon(s) is formed during exon skipping, which further undergoes internal processing to cut out the intron and create exonic circRNA [20]. This whole process starts with the 3′ end of an exon that joins to the 5′ end of the same exon or an upstream exon to generate exonic circRNA. (2) Intron-pairing circularization model: Most eukaryotic circRNAs are reported to be formed by the intron-pairing circRNA biogenesis model. In this model, base pairing between the flanking introns of the circularizing exons forms a stem–loop structure that brings the splice sites closer to favor backsplicing [21,22]. The inverted repeat sequences responsible for circRNA biogenesis mainly belong to Alu transposable elements. (3) RBP-mediated: In RBP-driven circularization, RBPs interact with the flanking introns and the circularizing exons to modulate the backsplicing of circRNAs [23]. Several *trans*-acting factors have been reported to interact with the flanking introns and regulate circular RNA biogenesis (Figure 1, Table 1). Here, we discuss the current knowledge of RBPs that are known to regulate circRNA biogenesis.

**MBL/MBNL1:** Flanking intronic sequences around exon 2 of the *Mbl* gene contain multiple binding sites for MBL/MBNL1 proteins, promoting circularization of exon 2 via the formation of the stem–loop structure bringing the splice sites to proximity [23]. This backsplicing event leads to the formation of *circMbl,* which can also act as a sponge for MBL proteins and modulate the linear splicing of *Mbl* pre-mRNA [23].

**QKI:** Quaking (*QKI*) is a group of three isoforms, *QKI-5, QKI-6,* and *QKI-7*, out of which the nuclear *QKI-5* is highly abundant and upregulated in epithelial–mesenchymal transition (EMT). QKI-5 binds to the QKI motif sequences in the flanking intronic sequence of circularizing exons, which promotes *circSMARCA5* formation during EMT [24]. In cardiac tissue, QKI-5 positively regulates the circRNA expression from Ttn, Fhod3, and Strn3 and negatively regulates circArhgap32 expression [25].

**HnRNPs and SR Proteins:** Heterogeneous nuclear ribonucleoproteins (hnRNPs) and serine–arginine (SR) proteins are well known for their role in pre-mRNA splicing. In Drosophila, the biogenesis of 490-nucleotide long circRNA from the exon 2 of the Laccase 2 gene depends on the flanking introns on both sides of the exon 2 containing a pair of transposons and interaction of flanking introns with hnRNPs and SR proteins [26]. SR proteins such as SRSF1, SRSF6, SRSF11, and hnRNP such as Hrb27C suppress Laccase 2 circRNA biogenesis, while Hrb87F promotes circRNA formation [26].

**RBM20:** RBM20 is a well-known regulator of alternative splicing, which regulates circular RNA formation from the Titin gene in cardiac tissue. Loss of function mutation in RBM20 in hypertrophic cardiomyopathy (HCM) leads to the loss of circRNAs generated from I band (Ig and PEVK regions) and alternative splicing of Titin genes [27]. RBM20 binds to the flanking introns of the circularizing exons and promotes exclusion of these portions from the pre-mRNA, promoting circRNA formation. In RBM20 knockout mice and HCM patients with RBM20 mutation, circRNA biogenesis is impaired, and the circularizing exons are included in the titin mRNA [27].

**DHX9:** The nuclear RNA helicase DHX9 interacts with the inverted Alu repeats transcribed with the genes. Alu repeats are known to regulate circRNA biogenesis by forming the stem–loop structure, facilitating backsplicing. DHX9 silencing leads to the upregulation of circRNAs and their host genes [28]. DHX9 was also found to interact with Alu-associated RNA-editing enzyme adenosine deaminase acting on RNA (ADAR). Alu-associated DHX9 and ADAR co-depletion promote double-stranded RNA accumulation and circRNA biogenesis by backsplicing [28]. Another study suggested that phosphorylation of DHX9 in oxaliplatin treatment promotes the expression of oncogenic circRNA *CCDC66*, which induces chemoresistance [29].

**Sam68:** Recent work suggested that the Survival of Motor Neuron (*SMN*) gene contains the highest density of Alus, which is often repeated in inverted orientation [30]. Furthermore, these inverted Alu repeats inhibit the splicing of long introns in the *SMN* gene and promote circRNA biogenesis. Interestingly, these Alu repeats in the *SMN* gene are predicted to contain Sam68 binding sites, which was further confirmed by biochemical approaches [30]. Together, the interaction of Sam68 with intronic Alu repeats in the *SMN* pre-mRNA regulates *SMN* circRNA biogenesis.

**NOVA2:** The neuronal RBPs NOVA1 and NOVA2 are well-known regulators of alternative splicing. Nova2 knockout in mice leads to a global reduction in circRNA levels in the embryonic cortex, suggesting enhancement of circRNA biogenesis by NOVA2 [31]. Furthermore, reporter constructs and cross-linking and immunoprecipitation assays indicated that NOVA2 interacts with YCAY clusters in the flanking introns of circRNAs such as *circEfnb2* and promote their biogenesis [31]. Together, this study indicates the regulation of circRNA biogenesis by NOVA2 in the developing brain.

**SFPQ**: The splicing factor proline/glutamine-rich (SFPQ) is enriched around circRNA, and silencing of SFPQ alters the expression of a subset of circRNAs with distal inverted Alu elements and long flanking introns (DALI circRNAs) [32]. Interestingly, knockdown of SFPQ leads to increased intron retention along with abnormal splicing and altered transcription termination in transcripts with long introns. Since circRNA biogenesis is dependent on the accurate splicing of the upstream and downstream introns, SFPQ regulates the biogenesis of Alu-independent DALI circRNAs by maintaining correct splicing of long introns around the circularizing exons [32].

**NF90/NF110:** The interleukin 3 (*IL3*) gene produces two isoforms of double-stranded RBPs, NF90 and NF110. Flanking introns of circRNA exon sequences contain reverse complementary sequences facilitating circRNA biogenesis. The NF90 and NF110 bind to these matching intronic sequences and form double-stranded stem structures that promote backsplicing by bringing the splicing sites to proximity [33]. Moreover, exporting nuclear NF90 and NF110 to the cytoplasm during viral infection reduces the biogenesis of circRNAs in the nucleus.

**HNRNPL:** One of the crucial splicing factors, heterogeneous nuclear ribonucleoprotein L (HNRNPL) is involved in the regulation of alternative splicing and circular RNA formation in prostate cancer cells. HNRNPL binds to the flanking intronic regions of circularizing exon sequences and promotes their backsplicing and generation of circRNAs. In addition, HNRNPL also inhibits the biogenesis of a few circRNAs to a lesser extent without affecting the parental mRNA [34]. Together, circRNA expression positively correlates with HNRNPL expression.

**FUS:** The splicing factor FUS protein binds to the flanking introns of exon–exon splice junctions and regulates circRNA biogenesis in mouse embryonic stem cell-derived motor neurons (MN cells) and neural crest cell-derived neuroblasts (N2a cells) [35]. Interestingly, neuronal cells in brain tissue show a high level of alternative splicing and generate many circRNAs. Mutation in the nuclear localization signal of FUS leads to nuclear export and cytoplasmic aggregation in amyotrophic lateral sclerosis. N2a FUS knockout cells show abnormal circRNA biogenesis in both nuclear and cytoplasmic compartments compared to control cells.

**ESRP1:** The circular RNA BIRC6 (*circBIRC6*) shows higher expression in human embryonic stem cells (hESCs) and is known to maintain pluripotency by sponging miR-34a and miR-145, which are known to induce differentiation in hESCs [36]. However, the biogenesis of *circBIRC6* is regulated through splicing factor epithelial splicing regulatory protein 1 (ESRP1), which is regulated by OCT4 and NANOG. Upregulation of ESRP1 in hESCs is directly linked to the binding of OCT4 and NANOG to the promoter region of *ESRP1* and increased H3K27Ac modifications, thereby promoting expression of ESRP1 in hESCs. In addition, ESRP1 recognizes the three GGT-rich binding motifs present in the upstream and downstream flanking introns across the exon–exon splice site of circBIRC6 and promotes circularization [36].

**TNRC6A:** The *circ0006916* originated from the homer scaffolding protein 1 (*HOMER1*) gene inhibits cell proliferation by halting cell cycle progression at G1/S transition. The biogenesis of *circ0006916* is controlled by RNA binding protein TNRC6A [37]. Both *circ0006916* and the interacting TNRC6A are cytoplasmic and are downregulated in lung cancer cells. The flanking introns of *circ0006916* contain inverted reverse complementary sequence and ALU repeats where TNRC6A interacts and promotes intron pairing across the circularizing exons [37].

**RBM3**: The splicing factor RBM3 shows higher expression in hepatocellular carcinoma and is reported to help liver cancer cell proliferation and survival. Overexpression of RBM3 in hepatocellular carcinoma (HCC) positively correlates to levels of *SCD-circRNA 2* generated from the stearoyl-CoA desaturase (*SCD*) gene [38]. In HCC, upregulated RBM3 binds to the flanking regions of *SCD-circRNA 2* and promotes its biogenesis.

## 3. The Functions of circRNAs

**Figure 2:**
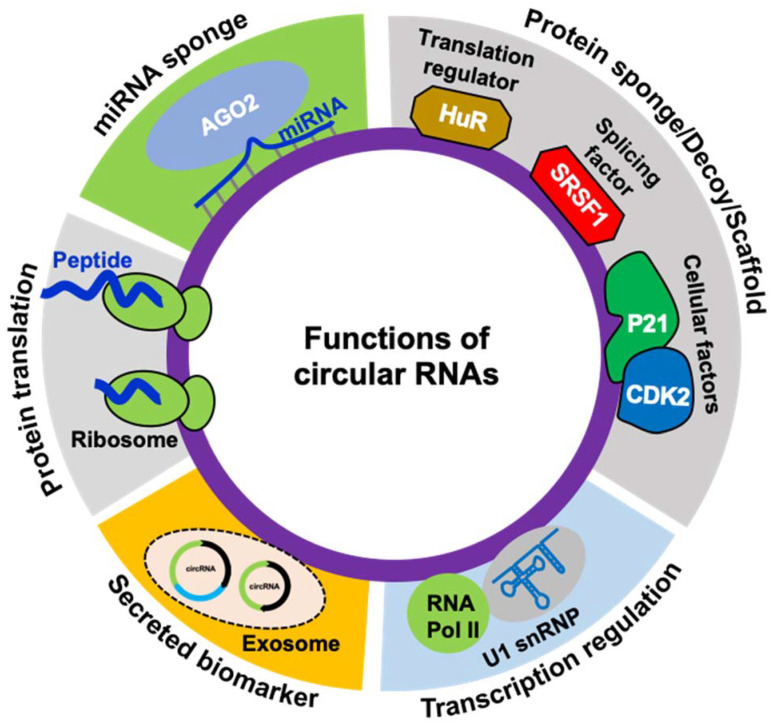
Schematic representation of known functions of circRNAs.

Experimental evidence suggests that circRNAs are critical regulators of human health and development. CircRNAs can regulate gene expression both at the transcriptional and post-transcriptional levels (Figure 2). Some of the ciRNAs and EIcircRNAs have been reported to localize into the nucleus and regulate nuclear events [39,40]. CiRNAs can modulate the transcription of the host genes by controlling the initiation and elongation of RNA polymerase II (RNAP II) [39]. PAR-CLIP analysis demonstrated that EIcircRNAs co-immunoprecipitate with RNAP II and regulate transcription [40]. Hundreds of studies have shown that circRNAs prominently localize in the cytoplasm and function as microRNA sponges, thereby regulating target gene expression [10,11,15]. CircRNAs with microRNA response elements (MREs) can inhibit the microRNA activity by sequestering it from binding to target mRNA, thereby increasing the expression of the downstream target genes. Probably the first and most studied circRNA that sponges miRNA is *CDR1as*, which possess about 70 conserved miR-7 binding sites [10,11]. Besides CDR1as, hundreds of circRNAs and their circRNA–miRNA–mRNA regulatory network have been reported and play a role in physiology [15,41,42]. Apart from miRNA sponging, another mechanism by which circRNAs can control gene expression is by interaction with RBPs. Many studies established that circRNAs can regulate RNA splicing and mRNA translation by acting as a sponge for RBPs [16]. For instance, *circMbl* biogenesis competes with *Mbl* mRNA linear splicing by sponging MBNL1 [23]. Another study reported that *circPABPN1* regulates PABPN1 translation by sponging the translation regulator HuR [43]. In addition, circRNAs regulate cellular function by acting as a scaffold for regulatory proteins. For example, *circFoxo3* serves as a protein decoy by interacting with cytoplasmic p21/CDK2 and forming a heterocomplex that impairs cell proliferation and promotes aging [44].

Exosomes are extracellular vesicles that primarily function as transport agents for diverse cellular components, supporting intracellular networking. Some recent studies observed the presence of stable circular RNAs in exosomes [45]. This phenomenon could further improve our understanding of exosomal circRNA, improving the use of circRNA for disease diagnosis and prognosis. Nearly a thousand exosomal circRNAs have been identified in the human body. Recent findings suggest that exosome-derived circRNAs may serve as diagnostic biomarkers against corresponding cancer depending on relative expression, stability, and exosome coupled targeted delivery route [46].

Most circRNA sequences arise from backsplicing protein-coding genes containing open reading frames (ORFs) and can act as templates for protein translation machinery (Figure 2). Since micropeptides translated from small ORFs are shown to be functional, translation of circRNAs into functional micropeptides or proteins remains to be explored [47,48]. Although circRNAs are primarily considered noncoding, several recent reports suggested the translation of circRNAs into functional proteins through cap-independent translation initiation mechanisms. CircRNAs with internal ribosomal entry sites and N-6 methyladenosine modifications associate with the translation initiation complex and translate into polypeptides [48,49,50]. For example, advanced mass spectrometry-based analysis and overexpression of circRNA plasmids confirmed the translation of two circRNA molecules, *circMbl* and *circZNF609*, both of which were shown to encode peptides [48,49]. However, the translation mechanism of circRNAs and the role of circRNA-derived proteins remains to be explored in detail.

## 4. The Function of circRNA–Protein Interactions

**Figure 3:**
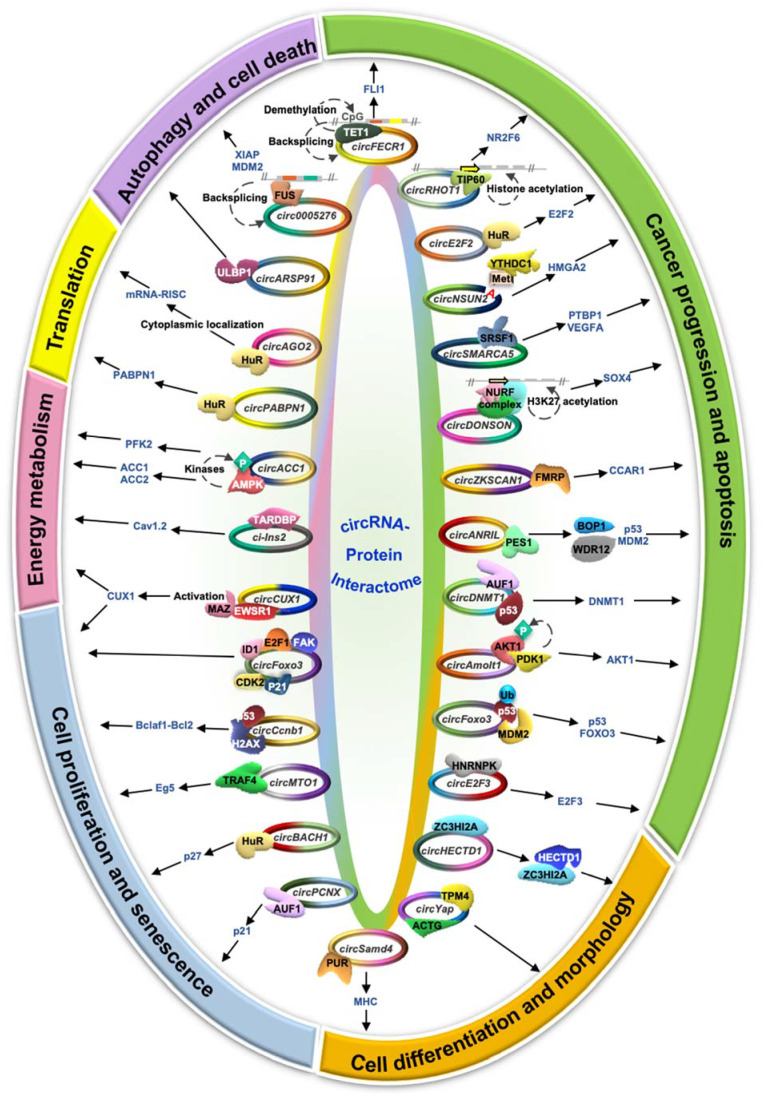
Schematic representation of circRNA–protein interactions and their implications in pathology and cellular physiological conditions.

Although the majority of the circRNA studies report that circRNAs regulate downstream genes by acting as miRNA sponges, several recent studies indicated the interaction of circRNAs with proteins that could further regulate downstream gene expression [15,16]. Hence, it is essential to understand the role of circRNA–protein interactions and their impact on gene expression and regulation. Circular RNAs are known to act as protein scaffolds to alter the function of the target protein, which modulates the downstream genes or pathways [16]. In addition, circRNAs act as recruiters for the associated proteins to specific locations to modulate gene expression. CircRNAs are also known to sponge many RBPs, and their sequestration leads to the altered expression of downstream RBP-target genes. Since circRNA–protein interaction has multipronged functional consequences in terms of gene expression and function, here we discuss the various mechanisms by which circRNAs regulate the function of the interacting proteins. Interestingly, circRNA–protein interactions regulate several aspects of cellular physiology such as cell proliferation, apoptosis, cancer cell metastasis, angiogenesis, mRNA translation, energy metabolism, and cell differentiation (Figure 3, Table 2).

### 4.1. Cancer Progression and Apoptosis

***CircZKSCAN1*:** The zinc-finger protein with KRB and SCAN domains 1 (*ZKSCAN1*) gene produces a 2232-nucleotide long *circZKSCAN1* (*hsa_circ_0001727*) by backsplicing of exon 2 and exon 3 [51]. The decrease in *circZKSCAN1* expression in hepatocellular carcinoma (HCC) tissues is correlated with increased cell migration, reduced apoptosis, and enhanced stemness of cancer stem cells. *CircZKSCAN1* acts as a sponge for FMRP (fragile X retardation protein), which positively regulates cell cycle and apoptosis regulator 1 (CCAR1) expression [51]. In addition, the CCAR1 protein activates the Wnt/β-catenin pathway in various cancers.

***CircSMARCA5*:***CircSMARCA5* (*hsa_circ_0001445*) generated from the *SMARCA5* gene is a 269-nucleotide long circRNA expressed in normal brain parenchyma. *CircSMARCA5* is highly downregulated in glioblastoma and further decreases with disease progression [52,53]. A decrease in the *circSMARCA5* expression leads to upregulation of the *circSMARCA5*-associated RBP serine- and arginine-rich splicing factor 1 (SRSF1), which promotes glioblastoma angiogenesis. Interestingly, *circSMARCA5* silencing leads to an increase in SRSF1 expression, promoting the expression of pro-angiogenic VEGFA and PTBP1 [52,53]. Hence, a decrease in *circSMARCA5* in glioblastoma enhances cell migration and angiogenesis by upregulating SRSF1 expression through PTBP1 and VEGFA.

***CircE2F2*:** The *E2F2* gene controlling cell proliferation and cell cycle progression gives rise to a 258-nucleotide long cytoplasmic *circE2F2* (*hsa_circ_0000030*) in ovarian cancer tissues and cell lines [54]. Silencing of *circE2F2* leads to decreased ovarian cancer cell proliferation, migration, invasion, and tumor cell growth compared to controls. In addition, the half-life of *E2F2* mRNA significantly decreases in the absence of *circE2F2*, indicating the role of *circE2F2* in maintaining the stability of *E2F2* mRNA [54]. *CircE2F2* interacts with HuR and regulates the stability of target *E2F2* mRNA.

***CircFECR1*:***FLI1* exonic circular RNA or *FECR1* (*hsa_circ_0000369*) originates from the exon 2-3-4 of friend leukemia virus integration 1 (*FLI1*) transcript found to be localized in both nuclear and cytoplasmic compartments [55]. Silencing of *FECR1* suppresses the rate of tumor invasion in metastatic breast cancer cells in the MDA-MB231 human breast adenocarcinoma cell line. *FECR1* induces *FLI1* transcription by promoting significant demethylation by recruiting TET1 demethylase to the promoter region [55]. *FECR1* downregulates DNMT1 (DNA methyltransferase1) expression by causing H3K27 acetylation in the promoter region of the *DNMT1* gene through unknown mechanisms.

***CircNSUN2*:***CircNSUN2* (*hsa_circ_103783*) is generated from exon 4 and 5 of the *NSUN2* gene and is upregulated in colorectal cancer patients and promotes tumor invasion and metastasis. The YTHDC1 binds to the methyladenosine modification on *circNSUN2* and promotes its nuclear export [56]. In the cytoplasm, *circNSUN2* interacts with IGF2BP2, which binds the 3′ UTR of *HMGA2* mRNA and promotes HMGA2 mRNA stabilization [56]. Together, the *circNSUN2*/IGF2BP2/*HMGA2* mRNA complex stabilizes HMGA2 mRNA to promote colorectal cancer metastasis.

***CircANRIL*:** Circular RNA *ANRIL* (*circANRIL*) generated from *lncANRIL* is expressed in human vascular tissues, smooth muscle cells, and macrophages. *CircANRIL* regulates ribosome biogenesis and cellular apoptosis by sponging pescadillo homolog 1 (PES1) protein, which interacts with blocker of proliferation 1 (BOP1) and WDR12 to form the PeBoW complex [57]. Since the PeBoW complex is critical for rRNA maturation, the interaction of *circANRIL* with PES1 inhibits PeBoW complex formation leading to inhibition of rRNA maturation and accumulation of ribosomal proteins such as L11, L5, and L23. These free proteins induce proteasomal degradation of MDM2, which leads to the stabilization of its direct target p53 [57]. Conclusively, *circANRIL* association with PES1 leads to p53 upregulation, increasing apoptosis in highly proliferating cancer cells.

***CircAmotl1*:***CircAmotl1* originates from exon 3 of parent gene angiomotin like-1 (*Amotl1*). Though *Amotl1* mRNA is highly conserved among species, the expression of *circAmotl1* is restricted to humans [58]. *CircAmotl1* interacts and activates AKT1 via phosphoinositide-dependent kinase 1 (PDK1-) dependent phosphorylation. Phosphorylated AKT (p-AKT) is translocated to the nucleus and promotes cell survival by inhibiting the expression of pro-apoptotic genes and suppressing cell survival genes [58].

***CircDNMT1*:** The exon 6 and 7 of DNA methyltransferase1 (*DNMT1*) genes gives rise to a circular RNA, *circDNMT1* (*hsa_circ_102439),* through backsplicing. *CircDNMT1* is upregulated in breast cancer cell lines and tumors compared to non-cancer cell lines and adjacent normal tissues, respectively [59]. *CircDNMT1* has binding sites for two RNA binding proteins such as AUF1 and tumor suppressor p53. AUF1 is known to reduce DNMT1 expression, and DNMT1 inhibits the apoptotic pathway by downregulating p53. Association of *circDNMT1* to AUF1 promotes nuclear localization of AUF1, which upregulates DNMT1 leading to inhibition of p53 expression [59]. In summary, increased expression of *circDNMT1* promotes breast cancer cell survival through the interaction of the circRNA with RNA binding proteins AUF1 and tumor suppressor p53.

***CircDONSON*:***CircDONSON* (*hsa_circ_0004339*) is a 948-nucleotide long circRNA generated from exon 3 to exon 8 of the *DONSON* gene. *CircDONSON* is upregulated in gastric cancer, promoting cell proliferation, migration, cell invasion, and inhibiting apoptosis through regulation of transcription of the Wnt signaling pathway gene SOX4 [60]. *CircDONSON* interacts with the NURF chromatin remodeler complex proteins SNF2L, BPTF, and RBBP4 and recruits it to the SOX4 promoter. Binding of the NURF chromatin remodeler to the *SOX4* promoter leads to H3K27 acetylation and H3K4 trimethylation promoting SOX4 transcription [60].

***CircRHOT1*:** The exon 3 to exon 6 of the *RHOT1* gene generates the nuclear-localized *circRHOT1* (*hsa_circ_0005397*) by backsplicing. *CircRHOT1* is highly expressed in hepatocellular carcinoma and regulates cell proliferation, cell migration, cell invasion, and inhibits apoptosis by modulating NR2F6 transcription [61]. In addition, *circRHOT1* binds to the -1000 to 800 bp region of the NR2F6 transcription start site and recruits TIP60. Recruitment of TIP60 promotes histone acetylation of the *NR2F6* promoter leading to upregulation of *NR2F6* transcription in hepatocellular carcinoma and correlates with poor prognosis [61].

***CircE2F3*:** The *E2F3* gene generates 720-nucleotide long cytoplasmic *circE2F3* (*hsa_circ_0075804*). *CircE2F3* is upregulated in retinoblastoma, promoting cell proliferation and inhibiting cell apoptosis [62]. The *circE2F3* sequesters HNRNPK and promotes its association with *E2F3* mRNA promoting *E2F3* mRNA stabilization [62]. Together, higher expression of *circE2F3* in retinoblastoma promotes *E2F3* mRNA stabilization leading to induced cell proliferation and reduced apoptosis.

***CircFoxo3*:** The *FOXO3* gene produces *circFoxo3* by backsplicing. *CircFoxo3* is minimally expressed in cancer cells and upregulated during cancer cell apoptosis. Ectopic overexpression of *circFoxo3* induces apoptosis in cancer cells, whereas silencing of endogenous *circFoxo3* promotes cell viability. Interestingly, *circFoxo3* binds to E3 ubiquitin ligase protein MDM2 and the tumor suppressor p53 [63]. Foxo3 and p53 are the known downstream targets of MDM2 for ubiquitin-mediated proteasomal degradation. This study suggested that *circFoxo3* inhibits Foxo3 degradation by promoting p53 ubiquitination by MDM2 [63].

### 4.2. Cell Proliferation and Senescence

***CircFoxo3*:** In addition to its role in cancer cell apoptosis, *circFoxo3* also controls cell proliferation by regulating G1-S phase transition by acting as a decoy for cyclin-dependent kinase 2 (CDK2) and the cell cycle inhibitor p21 [44]. During the mid-to-late G1 phase, CDK2 forms a complex with cyclin A/E and phosphorylates retinoblastoma (Rb) proteins. Phospho-Rb protein releases the E2F transcription factor that is responsible for the transcription of S phase-specific proteins. Since p21 inhibits the cell cycle progression by inhibiting CDK2 and cyclin A/E interactions, sponging of CDK2 and p21 protein by *circFoxo3* promotes cell division and viability [44]. Another study reported that *CircFoxo3* is upregulated in aged individuals compared to younger ones. *CircFoxo3* interacts with nuclear transcription factors and exports them to cytoplasm leading to inhibition of transcription factor functions. Cytoplasmic localization of *circFoxo3*-associated transcription factors, including ID1, E2F1, HIF1α, and FAK, leads to replicative senescence [64].

***CircBACH1*:** The *BACH1* gene generates a 1542-nucleotide long *circBACH1* (*hsa_circ_0061395*) from exon 3 and exon 4. *CircBACH1* acts as a sponge for HuR and promotes their cytoplasmic localization, where it binds to the 5′ UTR of p27 mRNA and inhibits p27 translation in HepG2 cells [65]. The protein p27 inhibits the cell cycle at the G1/S transition point, and low p27 expression increases cell proliferation via the accumulation of more cells in the S phase of the cell cycle. Thus, together, *circBACH1* promotes cell proliferation in hepatocellular carcinoma through cytoplasmic localization of HuR and inhibiting p27 expression [65].

***CircCcnb1*:** The nuclear-localized *circCcnb1* is generated from the exon 4 and 5 of the *CCNB1* gene. *CircCcnb1* is downregulated in metastatic breast cancer tissue. In normal tissue, *circCcnb1* binds to the H2AX protein that interacts with wild-type p53 leading to the interaction of free Bclaf1 to apoptotic inhibitor bcl2 resulting in cell proliferation [67]. However, in breast cancer, mutant p53 cannot bind to circCcnb1-H2AX leading to the formation of the circCcnb1-H2AX-Bclaf1 complex, which induces tumor cell death [67]. In addition, *circCcnb1* interacts with Ccnb1 and Cdk1 proteins known to form a complex and regulate mitosis. *CircCcnb1* dissociates the Ccnb1–Cdk1 complex and forms a large ternary complex of *circCcnb1*–Ccnb1–Cdk1, inhibiting tumor growth and extending survival [66].

***CircMTO1*:** The mitochondrial translation optimization 1 (*MTO1*) pre-mRNA generates *circMTO1* (*hsa_circ_0007874*), which is upregulated with monastrol treatment in breast cancer cells [68]. Interestingly, *circMTO1* is found to localize in the cytoplasm in monastrol-resistant MCF-7R cells, and *circMTO1* overexpression makes the cells sensitive towards monastrol. Moreover, silencing of *circMTO1* leads to an increase in cell viability of cancer cells, whereas overexpression leads to enhanced cell death and reduced cell viability. Thus, *circMTO1* sponges TRAF4 in the cytoplasm upon monastrol treatment and hence prevents the expression of Eg5, which blocks the bipolar spindle separation and ultimately inhibits proliferation of dividing breast cancer cells [68].

***CircPCNX*:***CircPCNX* (*hsa_circ_0032434*) is a 203-nucleotide long cytoplasmic circRNA generated from the *PCNX* gene [69]. *CircPCNX* acts as a decoy for the mRNA stability regulator AUF1 and promotes the stability of the downstream AUF1 target mRNAs, including *p21 (CDKN1A)* mRNA that encodes the cell cycle inhibitor p21. The sponging of AUF1 by *circPCNX* upregulates p21 expression, thereby suppressing cell proliferation in HeLa cells [69].

***CircCUX1*:** The *CUX1* gene gives rise to the 393-nucleotide long nuclear *circCUX1* (*hsa_circ_0132813*) that promotes cell proliferation, aerobic glycolysis, and tumor aggressiveness in neuroblastoma tumor cells [70]. *CircCUX1* interacts with EWS RNA binding protein 1 (EWSR1) and promotes its binding to MAZ that promotes MAZ activation [70]. Finally, the activated MAZ binds to the promoter region and activates the transcription of *CUX1* and other genes involved in cancer progression.

### 4.3. Energy Metabolism

***CircACC1*:** The *ACC1* gene generates the 383-nucleotide long *circACC1* from exon 2, 3, and 4 during metabolic stress or energy deprivation in the tumor microenvironment. *CircACC1* is cytoplasmic and regulates the assembly of the AMPK protein, which has α1, β1, and γ1 subunits. *CircACC1* binding to the AMPK leads to stabilizing AMPK by phosphorylation at the Thr 172 position through different kinases such as LKB1, CaMKK2, and TAK1 [71]. In addition, AMPK activation by *circACC1* leads to PFK2 phosphorylation that enhances ATP synthesis via glycolysis and ACC1 phosphorylation, leading to inhibition of fatty acid synthesis [71]. Together, serum starvation induces *circACC1* expression in colorectal cancer cells leading to activation of AMPK that promotes ATP generation via induction of fatty acid oxidation and glycolysis.

***Ci-Ins2*:** The conserved insulin gene in humans and insulin 2 gene in rodents generates circular RNAs from the intron 2 [72,80]. *Ci-Ins2* and its human ortholog *ci-INS* are downregulated in type 2 diabetes, predominantly localized in the nucleus, and interact with TARDBP [72]. TARDBP controls insulin secretion through the regulation of the expression of calcium channel protein Cav1.2. Silencing of *ci-Ins2* and TARDBP in rat islets leads to decreased insulin secretion and exocytosis in rat pancreatic islets [72]. Hence, *ci-Ins2* has an essential role in insulin secretion through sponging of the RNA binding protein TARDBP and is partly responsible for a defect in insulin secretion in the case of type II diabetes.

### 4.4. Translation Regulation

***CircPABPN1*:** The poly A binding protein nuclear 1 (*PABPN1*) gene generates a 152-nucleotide long circRNA known as *circPABPN1* (*hsa_circ_003188*) [43]. *CircPABPN1* regulates the translation of its parent *PABPN1* mRNA by sponging the RNA binding protein HuR. HuR binds to the 3′ UTR region of *PABPN1* mRNA and enhances its translation [43]. In sum, competitive sequestration of HuR by *circPABPN1* indirectly inhibits the translation of *PABPN1* mRNA and thereby decreases the expression of PABPN1 proteins.

***CircAGO2*:** The Argonaute 2 (*AGO2*) gene gives rise to a novel 391-nucleotide long intronic circular RNA, *circAGO2* (*hsa_circ_0135889*) [73]. *CircAGO2* is upregulated in various cancers and positively correlated with the advanced stages, metastasis, proliferation, invasiveness, and aggressiveness of cancer. For example, in gastric cancer cells, *circAGO2* interacts with nuclear HuR, which facilitates its export to the cytoplasm, where HuR promotes target mRNA translation by competing and preventing binding of the miRNA–AGO2 RISC complex to the same target [73].

### 4.5. Cell Morphology and Differentiation

***CircHECTD1*:** As the name suggests, the 380-nucleotide long *circHECTD1* (*mmu_circ_0000375*) is generated from the HECT domain E3 ubiquitin–protein ligase 1 (*HECTD1*) gene and expressed in the alveolar macrophages of lungs [74]. Chronic exposure to SiO_2_ downregulates the level of *circHECTD1* and upregulates the level of HECTD1 proteins. In healthy cases, *circHECTD1* sequesters ZC3HI2A in the cytoplasm, protecting it from degradation by HECTD1 that acts as an E3 ubiquitin ligase. At the same time, downregulation of *circHECTD1* enables ZC3HI2A to be free in the cytoplasm leading to its degradation by HECTD1 [74]. Thus, regulating the ZC3HI2A level through the *circHECTD1*/HECTD1 axis is essential in developing fibrosis upon chronic SiO_2_ exposure.

***CircSamd4*:***CircSamd4* is a 519-nucleotide long conserved circRNA that originated from the *Samd4* gene and is upregulated in differentiated myotubes compared to proliferating mouse C2C12 myoblasts. *CircSamd4* silencing reduced the expression of myogenic factors and delayed myogenesis [75]. Interestingly, *circSamd4* is associated with myogenesis repressor PUR proteins that inhibit myosin heavy chain (*Mhc*) transcription. This study suggested that *circSamd4* associates with PUR proteins preventing their interaction with Mhc promoters resulting in increased MHC expression seen during myogenesis [75].

***CircYap*:** The Yes-associated protein (*YAP*) gene generates three circRNAs (*circYap*) in human cardiac tissue with the highest expression levels of *hsa_circ_0002320*. *CircYap* is highly expressed in and downregulated in cardiac hypertrophy [76]. *CircYAP* inhibits the actin polymerization in cardiac tissue through binding and sequestration of tropomyosin 4 (TPM-4) and gamma actin (ACTG). Due to a decrease in *circYap* expression in cardiac disease, the formation of the *circYap*–TPM4–ACTG complex is hindered, leading to actin polymerization and cardiac hypertrophy [76].

### 4.6. Autophagy and Cell Death

***CircARSP91*:** Circular RNA *ARSP91* (*circARSP91; hsa_circ_0085154*) originates from the *PABPC1* gene. *CircARSP91* is downregulated in hepatocellular carcinoma cell lines and patient samples compared to healthy individuals [77]. ADAR1 converts adenosine to inosine, leading to reduced stem–loop formation inhibiting circRNA biogenesis. Consistent with this theory, higher expression of ADAR1 in HCC patients negatively correlates with the expression of *circARSP91* [77]. Furthermore, *circARSP91* interacts with UL16 binding protein 1 (ULBP1) in the HCC cell line leading to activation of the natural killer cells (NK cells) and increase in the tumor cells’ susceptibility to NK cells [78].

***Circ0005276*:***Circ0005276* originated from the X-linked inhibitor of apoptosis (*XIAP*) gene and binds the transcription factor FUS and promotes *XIAP* mRNA transcription [79]. Both XIAP and *circ0005276* show the highest expression in prostate cancer patients compared to healthy individuals. XIAP acts as the most potent autophagy inhibitor by ubiquitinating initiator caspases such as caspase 9 and effector caspases such as caspase 3 and caspase 7 [79]. In addition, XIAP targets apoptotic pathways by ubiquitinating negative regulators of p53 and MDM2 and enables its degradation via the proteasome pathway [81]. In sum, upregulation of *circ0005276* in prostate cancer promotes expression of XIAP, which induces proteasomal degradation of MDM2, leading to inhibition of autophagy and an increase in cancer progression.

## 5. Approaches to Analyze circRNA–Protein Interactions

Since the interaction of circRNAs with proteins regulates various cellular processes and regulates disease development, accurate identification of circRNA–protein complexes is critical for understanding their biological functions. Many biochemical methods have been developed to analyze RNA–protein interactions. However, only a few of them have been employed to study circRNA–protein complexes and their functions. The ribonucleoprotein immunoprecipitation (RIP) assay uses antibodies against circRNA-associated proteins, capturing the ribonucleoprotein complexes in the physiological lysis conditions. Subsequently, high-throughput RNA-seq, microarrays, or qPCRs of the RNAs captured in the RIP can be used to identify the interacting circRNAs [43]. Since RIP is performed at native conditions, it can detect RNAs indirectly associated with the target protein. Different versions of crosslinking and immunoprecipitation (CLIP) assays such as PAR-CLIP, HITS-CLIP, eCLIP, and iCLIP have been developed to identify RNA molecules directly associated with target proteins [82]. All these CLIP assays rely on crosslinking of the RBP with the interacting RNAs followed by RNase treatment to degrade the unbound RNAs, followed by immunoprecipitation and RNA-seq of the RBP-protected fragments. The physiological circRNA–RBP interaction can be validated by pulldown of the target circRNA by biotin-labeled antisense oligo complementary to the backsplice sequence followed by identifying interacting proteins using Western blot analysis [43,75,83]. Similarly, affinity pulldowns of in vitro synthesized biotin-labeled linear sequence of the circRNA followed by Western blotting or mass spectroscopy can identify the circRNA-associated proteins. Another method called the RNase protection assay (RPA) uses an array of complementary oligonucleotide DNA probes targeting circRNA sequences that induce RNase H-mediated degradation of unprotected circRNA sequences, leaving the circRNA sequences protected by RBP [84]. Hence, RBP binding sites can be found on circRNA sequences that have been protected from RNase H activity. In addition, the electrophoretic mobility shift assay (EMSA) helps analyze the interaction of RBP with *in vitro* synthesized RNAs containing radioactive- or fluorescent-labeled nucleotides [85]. The EMSA relies on the principle of the slow movement of RNA–protein complexes in a native PAGE compared to the unbound RNAs, visualized by radioactive or fluorescence approaches. Unlike the above techniques, the immune-histochemistry and fluorescence in situ hybridization (immune-FISH) assay makes it possible to visualize circRNA–RBP interactions in the cellular context [61]. Fluorescence signals from FISH probes targeting the backsplice junction sequence of target circRNAs along with fluorescence signals from antibodies used against circRNA-associated proteins can help in the visualization of circRNA–RBP complexes [61].

The advancement of high-throughput sequencing and deep learning-based bioinformatics tools has made it possible to predict circRNA–RBP interaction with accuracy. Some of the recent computational tools, including CRIP, circSLNN, DeCban, RPISeq, RPI-Pred, and RBPmap have been developed to identify RNA–protein interactions [86,87,88,89,90,91]. CRIP uses codon-based encoding and hybrid neural networks such as the convolutional neural network and recurrent neural network for the prediction of circRNA–RBP interaction along with RNA sequences input [86]. CircSLNN predicts RBP binding sites and specific sequence locations on circRNA based on the sequence labelling neural network model [87]. DeCban uses a double-embedding neural network and cross-branch neural network to identify RBP binding sites on full-length circRNA sequences [88]. RPISeq uses primary structure information to identify RBP binding sites and is based on a support vector machine (SVM) and random factor (RF) model [89]. RPI-Pred also works with the SVM approach for the identification of ncRNA–protein interaction [90]. RBPmap can be used to identify the RBP binding sites based on the RBP motifs present on the circRNA sequence [91]. Besides these computational prediction tools, web servers such as CircAtlas, starBase, and CircInteractome have been developed to identify RBP binding sites on circular RNA sequences using CLIP-seq datasets from various sources [8,92,93]. Computational prediction followed by validation by one or more of the biochemical assays described above will improve understanding of functional circRNA–protein interactions.

## 6. Concluding Remarks

CircRNAs have attracted much attention and have become a major topic of research in the last decade. With the availability of next-generation RNA sequencing technologies and new bioinformatics analysis tools, more than a million circRNAs have been identified in humans [8]. Interestingly, circRNAs are ubiquitously expressed in eukaryotes, from yeast to humans [94]. Thus, circRNAs have emerged as the novel regulator of cell physiology. CircRNA functions include regulation of transcription, mRNA stability, and translation by modulating the functions of microRNAs and RBPs [95]. RBPs are critical regulators of cellular gene expression that regulate the splicing, transport, modification, stability, and translation of transcripts. Realizing the critical role of circRNA interaction with RBP is advancing the knowledge on the mechanisms that control gene expression [16]. Several recent studies have increased our knowledge about the importance of circRNA–RBP interaction and given rise to a new direction for circRNA research. With the plethora of RNA–protein interaction sites identified with high-throughput CLIP-seq data sets, there is an urgent need to prioritize the circRNA–RBP interactions [8,93].

In this review, we have discussed the current knowledge on circRNA–RBP interactions and their functional implications in circRNA biogenesis and gene regulation. However, the analysis of circRNA–RBP interaction faces many challenges that need to be addressed: (1) The accurate prediction of mature circRNA sequences from RNA-seq is challenging. Hence, circRNA enrichment must be performed before RNA-seq library preparation, and specific circRNA annotation tools (e.g., CIRCexplorer2, circAST, CIRI-full) may be used to derive the mature spliced sequence [96,97,98]. In addition, circRNA sequences may be verified experimentally via circRNA-RCA before predicting the interacting RBPs [99]. (2) The second challenge is finding the RBP binding sites on circRNAs. Since circRNA sequences overlap with the parent mRNA and all CLIP-seq are performed on whole cell, finding the exact binding site of RBPs on circRNAs in the CLIP-seq data is challenging. Thus, novel CLIP-seq methods and computational tools need to be developed to improve the accuracy of finding circRNA–RBP interactions [8,93]. In addition, circRNA pulldown with biotin-labeled oligos needs to be improvised to identify the RBPs interacting with a circRNA in physiological conditions [43,75]. (3) The third challenge is the lack of bioinformatics tools or databases to predict functional RBP interaction with a given circRNA accurately. In addition, the association of multiple RBPs to a single circRNA poses another challenge to select a specific RBP for functional analysis. Therefore, it is essential to develop computational tools to assist the selection of circRNA-associated RBPs for functional characterization. These improvements will enhance our ability to identify functional circRNA–protein interactions and their physiological relevance.

Collectively, although circRNA–protein interaction research is in its infancy, emerging functional and mechanistic studies indicate the significance of circRNA–protein interaction in normal physiology and disease development. Undoubtedly, more and more functional studies on circRNA–protein interactions will recognize their relevance in human health and disease.

## Figures and Tables

**Table 1:** RBPs regulating the biogenesis of circRNAs.

RBP(s)	Cell Type/Tissue	Regulated circRNA(s)	Reference
MBNL1	Drosophila S2 and HEK293T	*CircMbl*	[23]
QKI	Epithelial-mesenchymal transition	*CircSMARCA5*	[24]
	Cardiac tissue	CircRNA from titin and striatin	[25]
HnRNPs and SR Proteins	S2 and DL1 cell culture lines of Drosophila	Laccase 2 circRNA	[26]
RBM20	Cardiac muscle tissue	CircRNAs from I-band of titin gene	[27]
DHX9	Human embryonic stem cells	CircRNAs with Alu element in the flanking introns	[28]
	Colorectal cancer	*CircCCDC66*	[29]
Sam68	HEK293T	*SMN* circRNA	[30]
NOVA2	Brain cortex	*CircEfnb2*	[31]
SFPQ	HepG2 and K562	DALI circRNAs	[32]
NF90/NF110	HeLa	*CircDHX34*	[33]
HNRNPL	Prostate Cancer LNCap	More than 200 circRNAs	[34]
FUS	N2a	More than 200 circRNAs	[35]
ESRP1	Human embryonic stem	*CircBIRC6*	[36]
TNRC6A	Lung cancer cell	*Circ0006916*	[37]
RBM3	hepatocellular carcinoma	*SCD-circRNA2*	[38]

**Table 2:** Functions of circRNA–protein interactions. ↑ represents upregulation and ↓ represents inhibition.

circRNA	Cell/Tissue, Localization	Cellular Localization	Interacting Proteins	Activity	Ref(s).
*CircZKSCAN1*	HCC	Cytoplasm	FMRP	Cancer cell stemness↑	[51]
*CircSMARCA5*	Glioblastoma	-	SRSF1	Angiogenesis and cell migration↓	[52,53]
*CircE2F2*	Ovarian cancer	Cytoplasm	HuR	Cell migration↑	[54]
*CircFECR1*	Breast cancer tissue	Nucleus and cytoplasm	TET1 demethylase	Metastasis↑	[55]
*CircNSUN2*	Colorectal cancer	Cytoplasm	YTHDC1	Metastasis↑	[56]
*CircANRIL*	Atherosclerosis	-	PES1	Apoptosis↑	[57]
*CircAmotl1*	Heart	-	AKT1	Apoptosis↓	[58]
*CircDNMT1*	Breast cancer	Nucleus	AUF1	Apoptosis↓	[59]
*CircDONSON*	Gastric cancer	-	NURF complex	Apoptosis↓	[60]
*CircRHOT1*	HCC	Nucleus	TIP60	Apoptosis↓	[61]
*CircE2F3*	Retinoblastoma	Cytoplasm	HNRNPK	Apoptosis↓	[62]
*CircFoxo3*	Breast cancer	Cytoplasm	p53, MDM2	Apoptosis↑	[63]
*CircFoxo3*	Cardia fibroblast	Cytoplasm	p21, CDK2	Cell proliferation↓	[44]
*CircFoxo3*	Heart	Cytoplasm	ID1, E2F1, HIF1α, FAK	Replicative senescence↑	[64]
*CircBACH1*	HCC	Nucleus and cytoplasm	HuR	Cell proliferation↑	[65]
*CircCcnb1*	Breast cancer	Nucleus	H2AX	Cell proliferation↑	[66,67]
*CircMTO1*	Breast cancer	Cytoplasm	TRAF4	Cell proliferation↓	[68]
*CircPCNX*	HeLa	Cytoplasm	AUF1	Cell proliferation↓	[69]
*CircCUX1*	Neuroblastoma	-	EWSR1	Cell proliferation↑	[70]
*CircACC1*	Colorectal cancer	Cytoplasm	AMPK	Glycolysis and fatty acid oxidation↑	[71]
*Ci-Ins2*	Pancreatic β-cells	Nucleus	TARDBP	Insulin secretion↑	[72]
*CircPABPN1*	HeLa	-	HuR	PABPN1 translation↓	[43]
*CircAGO2*	Gastric cancer	Cytoplasm	HuR	Binding of RISC to mRNAs↓	[73]
*CircHECTD1*	Alveolar macrophage	Cytoplasm	ZC3HI2A	Macrophage differentiation↑	[74]
*CircSamd4*	C2C12 myoblast	Cytoplasm	PUR proteins	Myogenesis↑	[75]
*CircYap*	Cardiac fibrosis	Nucleus	TPM4 and ACTG	Actin polymerization↓	[76]
*CircARSP91*	HCC	-	ULBP1	Cell death by NK cells↑	[77,78]
*Circ0005276*	Prostate cancer	-	FUS	Autophagy↓	[79]
